# Transdisciplinary Online Health Assessment of an Artisanal and Small-Scale Gold Mining Community during the COVID-19 Pandemic in the Mandalay Region of Myanmar

**DOI:** 10.3390/ijerph182111206

**Published:** 2021-10-25

**Authors:** Win Thiri Kyaw, Yee Mon Myint, Xiaoxu Kuang, Masayuki Sakakibara

**Affiliations:** 1 Research Institute for Humanity and Nature, Kyoto 603-8047, Japan; xxkuang@chikyu.ac.jp (X.K.); sakaki@chikyu.ac.jp (M.S.); 2 Magway General Hospital, Magway 04011, Myanmar; yeemonmyint@gmail.com; 3 Graduate School of Science and Engineering, Ehime University, Matsuyama 790-8577, Japan

**Keywords:** artisanal and small-scale gold mining, online health assessment, Myanmar, coronavirus disease, mining community

## Abstract

Artisanal and small-scale gold mining (ASGM) has a known negative effect on the community’s health; therefore, assessment to monitor community health is essential to detect any issues and enable early treatment. Because ASGM-related health issues are complex and cannot be addressed effectively with a traditional one-time health assessment alone, both long-term and regular health assessments using a transdisciplinary approach should be considered. In response to this need, we designed an online health assessment tool as a reference for a future long-term health assessment system. An online video interview was conducted with 54 respondents living in the ASGM area of Chaung Gyi Village, Thabeikkyin Township, Mandalay Region, Myanmar, via a social networking service application. The tool was used to evaluate community health during the coronavirus 2019 pandemic, including mercury intoxication symptoms, mining-related diseases, and other diseases. Results show that persons working in mining versus non-mining occupations had a greater prevalence of pulmonary diseases, such as pulmonary tuberculosis, silicosis, and bronchial asthma, in addition to malaria. Based on these findings, online health assessment using a transdisciplinary approach can be recommended as an effective tool for sustainable and long-term health assessment of ASGM-related disease and should be performed regularly following physical health surveys.

## 1. Introduction

Artisanal and small-scale gold mining (ASGM) is a major livelihood of rural communities in developing countries; however, ASGM is also widely known as a major source of atmospheric pollution by mercury (Hg) [1] and other heavy metals, as well as the cause of serious health problems in mining communities. Elementary Hg is used in the ASGM process to extract gold (Au) from ore, and the Au-Hg amalgam is further burned to obtain Au, which releases Hg into the atmosphere. Because some ASGM activities take place in residential communities without a proper containment system, the Hg vapor released during the amalgam-burning phase is widely dispersed, leading to Hg infiltration into the bodies of persons working in mining (hereafter, miners) and other local community residents through inhalation.

Several countries are focusing on reducing the use of Hg in the ASGM process, but Hg amalgamation remains a favored method to obtain Au because of its accessibility, affordability, and applicability in local settings. According to previous studies in ASGM areas internationally, Hg is a serious health hazard in ASGM workers, regardless of the origin [2,3,4,5,6,7,8,9,10,11]. In addition to Hg-related health issues, ASGM can also cause infectious diseases, pulmonary diseases, and accidents resulting from dust inhalation [12]. All these studies point out that regular health assessments of ASGM communities are essential in monitoring their long-term health.

However, several major limitations exist for regular health assessment in ASGM communities because of the nature of the industry. Typically, ASGM is performed in remote areas situated far away from the medical facilities available, and thus, the communities cannot easily receive critical health care. It is also challenging to conduct regular assessments of ASGM community health. In addition, health surveys in ASGM areas are usually performed either only once or in limited frequency, which cannot sufficiently contribute to the understanding of the health status of the community, especially because of the severe nature of the ASGM-related problems. Moreover, this dire situation has escalated during the global pandemic of coronavirus disease (COVID-19) because onsite health surveys and in-person interactions are not possible.

Therefore, it is essential to develop effective and long-term sustainable health assessments of ASGM areas to monitor the community health status and solve related issues with a transdisciplinary approach. In this study, an online health survey was conducted in the ASGM community of Chaung Gyi Village, Thabeikkyin Township, Mandalay Region, Myanmar, during the COVID-19 pandemic. A video health interview was conducted using a transdisciplinary approach with collaboration among researchers, the local physician, the government, and local stakeholders as a case study for designing a future, long-term, sustainable health assessment. According to the findings of our previous preliminary research in the study area, a small number of miners had the signs and symptoms of chronic Hg intoxication [13]. This case study reports the health status of the ASGM community and discusses the effectiveness of the collaborative online health assessment tool.

## 2. Materials and Methods

### 2.1. Study Area

**Figure 1:**
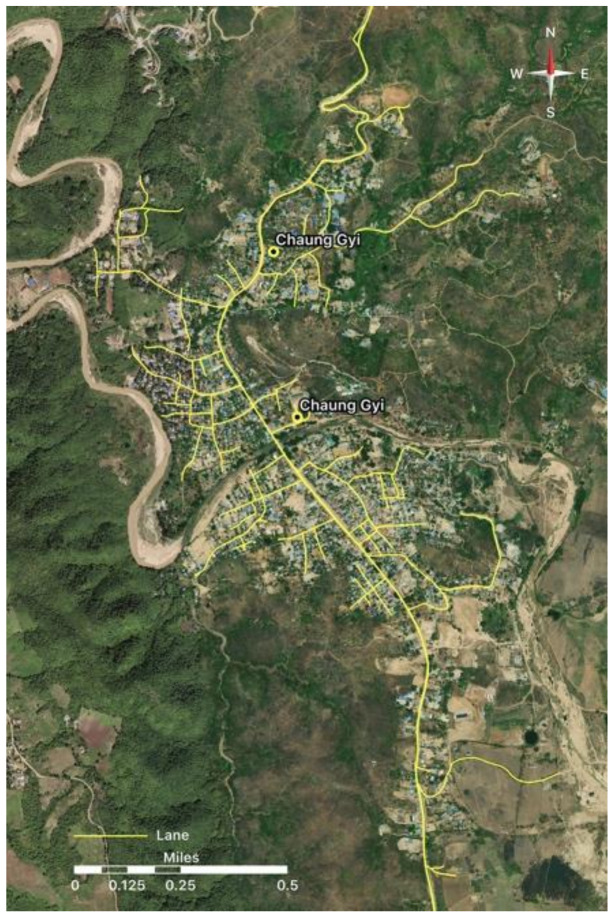
Study area. Map of Chaung Gyi Village. (Map is provided by Network Activities Group (NAG), a local NGO of Myanmar).

The location of the Chaung Gyi Village is depicted in Figure 1. According to the March 2019 data of the administrative office, Chaung Gyi Village has 1772 households and a population of 8375 people. The ASGM activity of Chaung Gyi Village can be classified into formal and informal types [13].

### 2.2. Transdisciplinary Approach Research Design

**Figure 2:**
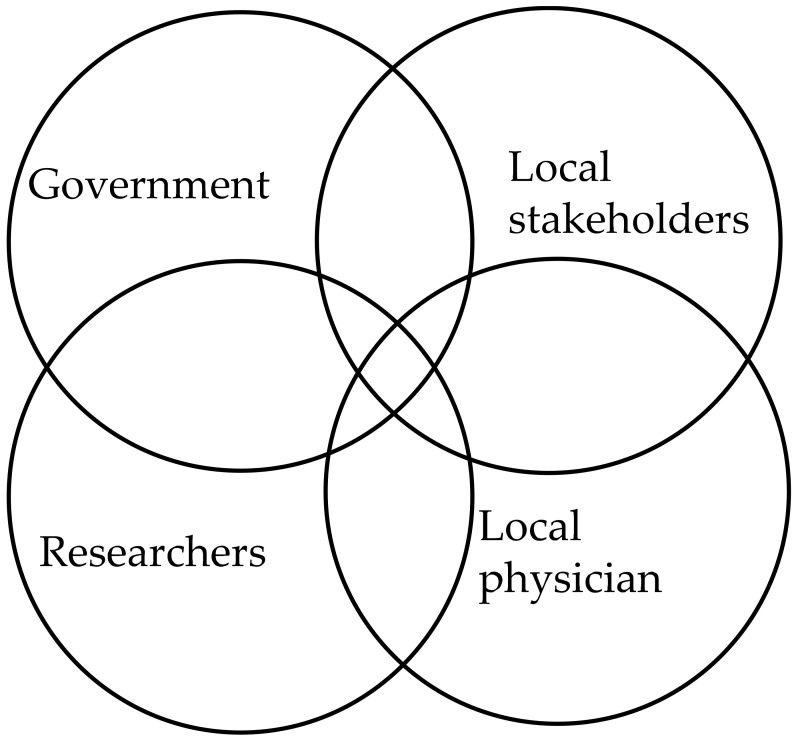
Transdisciplinary approach research design.

Between December 2020 and February 2021, 54 individuals from Chaung Gyi Village, Chaung Gyi Village Tract, Thabeikkyin Township, Mandalay Region, Myanmar, were recruited for this study. The mean age of the participants was 36.8 ± 13.4 years; among them, 32 were male, and 22 were female. As shown in Figure 2, Myanmar government entities, including the Environmental Conservation Department (ECD) of the Ministry of Natural Resources and Environmental Conservation (MONREC), and the local stakeholders of Chaung Gyi Village, researchers, and local physicians worked together and contributed to the implementation of the online health assessment. The ECD and MONREC shared ASGM information for the study area with researchers through collaboration with local stakeholders, and the local stakeholders contributed their support to connect the ASGM community of Chaung Gyi Village with the researchers and the local physician. Then, the researchers and local physicians contributed to the design and development of the online health assessment tool to make it suitable for the study area through collaboration with the government and local stakeholders, including the mining community. Then, the researchers and local physicians engaged the community (hereinafter referred to as “respondents”) in a conversation by video interviews of the respondents’ social networking service application to explain and conduct the study. Verbal informed consent was obtained from the respondents because the nature of the study made it impossible to receive their written consent.

### 2.3. Interview Questions

Respondents used the questionnaire form to provide the following data: (1) general information, including living status as a native to the study area or migrant, education, occupation, and income; (2) risk of exposure to Hg and other heavy metals, such as the distance of their homes to ASGM sites, use of Hg and cyanide in the ASGM process, and use of personal protection for miners; (3) health complaint; (4) present and past medical histories; (5) symptoms of Hg intoxication, such as metallic taste, excessive salivation, tremors, sleep disturbances, tiring easily, feeling sleepy or drowsy, lack of energy, concentration problems, forgetfulness, feeling nervous or sad, palpitations, headaches, nausea, loss of appetite, weight loss, numbness, prickling, and aching sensations. Then, during the video interview, respondents were evaluated for signs of Hg intoxication, gait ataxia, tremor on the finger-to-nose test, and alternate movements on the twist-hand test. Based on their type of occupation at the time of health assessment, respondents were divided into two groups: miners and non-miners.

### 2.4. Statistical Analysis

An unpaired *t*-test was employed for comparison of the mean values, and Fisher’s exact test was performed for the analysis of comparison among the variables of miners and non-miners, respectively. The level of significance was set at *p* < 0.05.

## 3. Results

### 3.1. Socioeconomic Assessment

Table 1 describes the results of the demographic and socioeconomic assessments. The number of miners was 24 (male; 18 and female 6), and that of non-miners was 30 (male 14 and female 16). The percentage of male and female participants and age were equally distributed. Most participants were aged 20–29 years. No difference was noted between the two groups in terms of living status as native or migrant and education status. Most respondents had completed primary education. The income of the miners ranged from $6–$31 USD/day, which was considered to meet the threshold of monthly family expenses, whereas those in local non-mining occupations earn less (*p* = 0.01). Some of the miners of this study work in the underground mines, 20–30 m below the surface of the ground, whereas some work to retrieve gold using the sluicing methods in the rivers. Miners who work for the excavation of ores underground mostly work long continuous hours for one to two days at the mining site. The underground mining area is situated far from the village, from where the excavated Au-containing ore is usually transported to the village for further processing, such as crushing, screening, and refining at the homes of miners, local gold shops, or in open spaces on the streets of the village. Crushing ore is done using simple equipment in front of their houses, and then screening ore is made in the reservoir dug by the miners in the yard. The Au-amalgam is prepared by mixing the Au-containing ore with the Hg purchased from local convenience stores, after which the amalgam is burned to evaporate the Hg into the atmosphere and retrieve the Au. This process is known as refining.

The categories of occupants in the non-miner group were agriculture, bricklaying, vehicle driving, buying and selling goods, miscellaneous jobs, and household jobs. Three men from the non-miner groups worked previously at random in excavation sites and in crushing ores of mining temporally, but not as the occupation. The respondents engaged in agriculture were the owners, or the owners’ family members, of the lands where they were working, and their working routine varied according to the seasons and types of crops and vegetables they were growing. The agricultural products produced were consumed by the producers as well as sold in the local wet markets and other distant cities. The bricklaying category of occupation was irregular and dependent on the availability of the job offer. The earnings from buying and selling of goods also depended on the demand of the consumers, which is similar to the nature of miscellaneous jobs and household jobs in the aspect of income being unstable. Compared with these non-mining jobs, mining work contributed to a more stable income in the study area.

### 3.2. Risk of Exposure to Hg and Other Heavy Metals

The factors influencing exposure to Hg and other heavy metals are detailed in Table 2. Of the 54 respondents, 25.9% (*n* = 14) reported living at a distance less than 1 km from ASGM activity. Most miners used Hg in their ASGM activities. Moreover, more than two-thirds of the miner group did not use personal protection, such as gloves and masks. These factors represent a high possibility of exposure to Hg and other heavy metals. Regarding the types of mining activities, most miners worked in excavation and crushing ores. In addition, most miners had worked in the ASGM occupation for 10–20 years.

### 3.3. Disease Prevalence by Occupation

**Figure 3:**
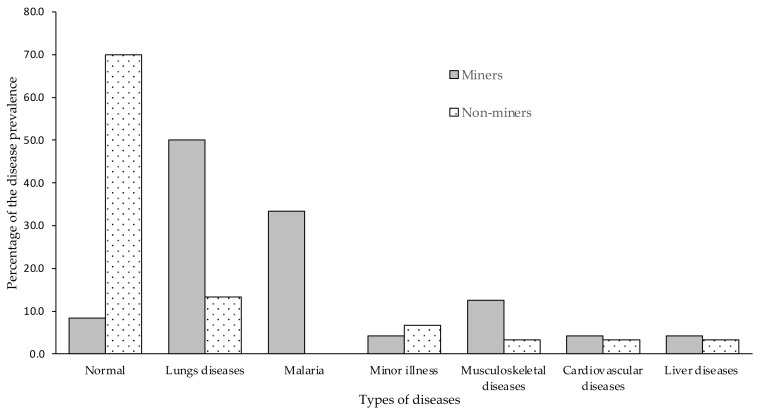
Disease prevalence in miners and non-miners.

Health complaints, including present and past medical histories of the respondents, were summarized as the disease prevalence, as shown in Figure 3. According to the interview results of the respondents, disease prevalence was classified into “normal” representing no health issues; “lung diseases” with pulmonary health issues; “malaria;” “minor illness” with minor health complaints; “musculoskeletal diseases,” problems in joints, bones, muscles, and spine; “cardiovascular diseases” having typical symptoms of cardiovascular diseases including pain or pressure in the chest, pain or discomfort in the arms, left shoulder, elbows, jaw, or back, shortness of breath, nausea and fatigue, lightheadedness or dizziness and cold sweats; and “liver disease” which includes jaundice, abdominal pain and swelling, nausea, vomiting, and melaena.

On comparison of miners versus non-miners, 50% (*n* = 12) of miners, especially those who engaged in excavation and crushing ores, had greater pulmonary issues, including pulmonary tuberculosis, silicosis, and bronchial asthma, versus 13.3% (*n* = 4) of the non-miner group, which had pulmonary tuberculosis and bronchial asthma (*p* = 0.01). Miners with mining experience greater than 10 years had pulmonary disease, and they had to reduce their workload due to their health problems. History of malaria infection was also only detected in the miner group (*n* = 8, 33.3%). The prevalence of musculoskeletal diseases was 12.5% (*n* = 3) for miners and 3.3% (*n* = 1) for non-miners. The prevalence of cardiovascular diseases and liver diseases for miners were 4.2% for each category (*n* = 1) and non-miners were 3.3% for each category (*n* = 1) respectively. 

### 3.4. Chronic Hg Intoxication Signs and Symptoms

Of the miners, 19% reported symptoms of Hg intoxication, such as sleep disturbance, loss of appetite and weight, depression, and numbness of the digits. Non-miners did not have any symptoms. However, no abnormal findings on the examination for the signs of Hg intoxication were noted for either group.

## 4. Discussion

### 4.1. Factors in the Choice of ASGM as the Main Livelihood in the Study Area

According to the nature of the socioeconomic characteristics, it can be clearly noted that ASGM can contribute to a stable and higher income for individuals within the community who have the same educational background compared with those in the non-mining occupations available in the study area, such as agriculture and other job options. Because ASGM activities can be conducted within the study area at a close distance to the community dwelling places, the accessibility to the ASGM areas is high. Therefore, ASGM may be the preferred occupation in the study area for the aforementioned factors, combined with the context of regional poverty.

### 4.2. Community Health Status

The study area community had a significant incidence of pulmonary disease, including pulmonary tuberculosis, silicosis, and bronchial asthma, in addition to malaria and musculoskeletal, cardiovascular, and liver diseases. Compared with the non-miner group, the miner group had an increased incidence of pulmonary diseases and malaria. Symptoms of pulmonary diseases, such as silicosis and pulmonary tuberculosis, have been reported by ASGM miners who worked in excavating and crushing ores as a major ASGM activity; however, these symptoms have not been detected in miners who performed only panning, amalgamation, or burning amalgam. The risk for silicosis is caused by inhalation of silica crystalline, which is a common health hazard in mining and increases the risk for silicosis, malignancy, and other diseases [14]. Silica exposure is related to the development of various diseases, including pulmonary tuberculosis, chronic obstructive pulmonary disease, and rheumatoid arthritis [15]. Therefore, the cause of the high incidence of pulmonary tuberculosis in miners in this study may be due to the presence of silicosis, which can also cause secondary infection in the non-mining community, such as in the families of miners. Because Myanmar is a developing country with poverty in remote areas, pulmonary tuberculosis is common. Therefore, it is necessary to decrease the incidence of ASGM-related silicosis to relieve the national burden of pulmonary tuberculosis. Malaria is prevalent in the Thabeikkyin Township [16], and the miners’ nature of working in the mines and sleeping without sleeping nets could have led to increased exposure to malaria, in the present study, relative to that among the non-miners.

Regarding the health hazard of Hg exposure, only 19% of the miners had the symptoms of chronic Hg intoxication, although most of the miners were exposed to Hg through the ASGM process. Similarly, in our previous preliminary study, we detected the signs and symptoms of chronic Hg intoxication in only a minority of the miners, ref. [13] and Hg concentration in their hair was not high compared with that in ASGM areas of other nations. Considering these factors together, it can be assumed that the major health problem in the study area mining community is silicosis and its associated complications rather than chronic Hg intoxication. The reason for the absence of chronic Hg intoxication in the present study may be the lower Hg usage in the study area compared to that in other ASGM areas worldwide, which may have caused the decreased formation of methylmercury in the environment. However, it is difficult to draw conclusions only on this basis owing to the small sample size; thus, the continuous assessment remains necessary to evaluate the status of chronic Hg intoxication.

### 4.3. Effectiveness of Online Health Assessment in ASGM Context

To our knowledge, this study is the first to present an online health assessment conducted for the ASGM community during the COVID-19 pandemic. The researchers, local physicians, local government, and local stakeholders contributed equally to the study design during this difficult time of COVID-19, which limited access to the respondents. Being far from the health facilities, the unavailability of laboratory services to detect Hg concentrations in hair as biomonitoring data, and the limited ability to install the remote health monitoring systems in the study area results in the community remaining vulnerable to mining-related health hazards to date. However, applying this remote health assessment in the study area through video interviews using the individuals’ mobile phones enables preliminary health assessment, which can help facilitate the diagnosis of diseases in the mining community to decide on further necessary treatment and management. 

When compared to the past onsite preliminary study of the study area conducted during the non-COVID-19 pandemic period [13], the present study offers the following merits (1) shorter processing time for the survey investigators, (2) ability to adjust the interviewing time as per the availability of both the survey investigators and respondents, (3) ease of contacting the investigators by the researcher, local physician, and respondents, (4) greater chance to learn the lifestyles of the mining community that is important in disease prevalence, and (5) the ability to monitor the health of the mining community in a consecutive manner, which is a challenging aspect for onsite surveys. Despite these advantages, there are some disadvantages, including the lack of clinical examination and the lack of analysis of the biomarkers of Hg, such as in hair and blood Hg contents. Therefore, it is recommended that onsite health assessment surveys be conducted to evaluate clinical examinations and the biomarkers once every year, followed by regular multiple online health assessments to effectively manage the health of the mining community.

Our study also had some limiting factors, such as the small sample size and the aforementioned fact of being unable to conduct the physical examinations of the respondents in person. However, the design of the present study allows the health assessment of the study area through communication among the researchers, local physician, and community by means of an individual online connection with community members, through which we hope that we can also implement regular and long-term assessment of the ASGM community in the future, conceived as an “online assessment system for the ASGM community.” Furthermore, because the ASGM context reflects the severity of the associated health and environmental hazards, the traditional assessment using biomonitoring, such as the evaluation of Hg content in hair, is solely insufficient for the ASGM community health assessment. In the future, a trained local health assessment team, including the nurses and local stakeholders, for physical monitoring of the health of the local ASGM community should be formed with the support of physicians and researchers. As an adjunct to that team, the “online assessment system for the ASGM community” by health professionals and researchers in various fields is recommended for an effective management strategy to address the health-related problems of ASGM.

## 5. Conclusions

We present a case study of the effectiveness of the first online health assessment in an ASGM community in Myanmar, conducted with a transdisciplinary approach. The main health problems of the community of the study area were pulmonary disease and health-related complications due to the ASGM process. This assessment can be conducted serially and regularly in the future to monitor the health status of the ASGM community from various aspects in collaboration with local stakeholders, health professionals, and researchers.

## Figures and Tables

**Table 1:** Demographic and socioeconomic assessments.

Variable	Category	54 Respondents (%)	Miners	Non-Miners
Sex	Male vs. Female	59.3	18 vs. 6	14 vs. 16
Age (years)	Mean ± SD		37.3 ± 11.7	36.5 ± 14.2
Age (years)	20–29	38.9	9 (*n*)	12 (*n*)
	30–39	24.1	4 (*n*)	9 (*n*)
	40–49	16.7	6 (*n*)	3 (*n*)
	50–69	20.3	5 (*n*)	6 (*n*)
Living status	Native vs. migrant	59.3	13 vs. 11	19 vs. 11
Education status	Monastic School Education	7.4	1 (*n*)	3 (*n*)
	Primary School	51.9	8 (*n*)	20 (*n*)
	Middle School	16.7	7 (*n*)	2 (*n*)
	High School Completed	13	4 (*n*)	3 (*n*)
* Income per day (USD)	Mean ± SD		14.7 ± 10.8	2.5 ± 1.5
Income per day (USD)	Range (minimum–maximum)		$6–$31	$1–$8

* Statistically significant (*p* = 0.01) in unpaired *t*-test. Monastic school education contributes to the basic education need of the people with poverty who could not afford to attend regular schools and provide the curriculum education and ethics to the students.

**Table 2:** Risk of exposure to Hg and other heavy metals.

Risk Factor	Numbers (*n*)
Living distance from ASGM activity of all respondents	
<1 km	14
1–5 km	17
5–10 km	23
>10 km	0
Miner-Specific Risk Factor of miners	
Hg use	12
Cyanide use	3
Personal protection use during mining activity of miners	
Yes	9
No	15
Mining activities of miners	
Working across all areas of mining	6
Excavation and crushing ores primarily	10
Panning and amalgamation only	4
Refining (burning amalgam) only	2
Carrying gold ores only	2
Mining experience of miners	
<10 years	8
10–20 years	14
>20 years	2

## Data Availability

The data that support the findings of the study are available from the corresponding author (Kyaw, W.T.) upon reasonable request.
